# Endothelin-1 triggers oxidative stress and cytokine release in human microglia cells through ETRB-dependent mechanisms

**DOI:** 10.3389/fncel.2025.1677457

**Published:** 2025-09-23

**Authors:** Yaritza Inostroza-Nieves, Shakira Bou, José Alvarado, Diego Capo-Ruiz, Jessica Garcia, Jean P. Moliere, Claudia P. Arenas

**Affiliations:** 1 Department of Biochemistry and Pharmacology, San Juan Bautista School of Medicine, Caguas, PR, United States; 2 Universidad Autónoma de Guadalajara School of Medicine, Zapopan, Mexico

**Keywords:** endothelin, inflammation, microglia, reactive oxygen species, STAT1

## Abstract

Microglial cells are highly specialized cells of the central nervous system (CNS) that play dual roles in neuroprotection, but can also promote inflammation and neurodegeneration. Endothelin-1 (ET-1) is a potent vasoconstrictor that induces severe and prolonged cerebral vasoconstriction and inflammation. However, the mechanism of how ET-1 activates a proinflammatory response in the CNS is unknown. In this study, we demonstrate that ET-1 activates proinflammatory and oxidative stress responses in human HMC3 microglial cells via endothelin receptor B (ETRB). ET-1 treatment significantly increased nitric oxide (NO) and reactive oxygen species (ROS) production, and upregulated inducible nitric oxide synthase (iNOS) mRNA. These effects were attenuated by the selective ETRB antagonist BQ788, but not by the ETRA antagonist BQ123, suggesting a receptor-specific mechanism. ET-1 increases TNFα levels by 56% (*p* = 0.0003) and IL-6 levels by 86% (*p* = 0.0111), and the effect was decreased to basal levels in the presence of BQ788. Moreover, ET-1 induced phosphorylation of STAT1 (3.5 folds, *p* < 0.0001), a transcription factor associated with microglial proinflammatory polarization. To validate the *in vivo* relevance of this pathway, we analyzed brain tissue from experimental autoimmune encephalomyelitis (EAE) mice. We found increased expression of Edn1 and Ednrb, as well as elevated ET-1 protein levels. These results identify ET-1/ETRB signaling as a key driver of microglial activation and oxidative stress, highlighting its potential as a therapeutic target in neuroinflammatory disorders.

## Introduction

Microglia are the primary immune cells of the central nervous system (CNS), responsible for maintaining homeostasis, modulating synaptic activity, and responding to injury or inflammation through cytokine release and phagocytosis (Colonna and Butovsky, 2017; Merson et al., 2010). Upon activation, they produce proinflammatory cytokines, nitric oxide (NO), and reactive oxygen species (ROS), which can contribute to neuroinflammation and neurodegeneration (Kempuraj et al., 2016; Brown and Vilalta, 2015).

Endothelin-1 (ET-1) is a potent vasoactive peptide expressed by endothelial cells, astrocytes, and neurons in the brain. It exerts its effects through two G-protein coupled receptors, ET_A (ETRA) and ET_B (ETRB), and is known to regulate vasoconstriction, inflammation, and blood–brain barrier integrity (StatPearls, 2023; Koyama, 2021). ET-1 has been implicated in the progression of various neurovascular and neurodegenerative diseases (Nie and Olsson, 1996; Schinelli, 2002; Faraco et al., 2013) but its direct role in microglial activation is unclear.

In this study, we investigated the role of ET-1 in the activation of human microglial cells. Using the HMC3 cell line, we evaluated the production of proinflammatory cytokines (TNF-*α* and IL-6), NO, ROS, and the activation of STAT1 signaling in response to ET-1 and explored the contribution of ETRB to these effects. To bridge our mechanistic findings with disease relevance, we further evaluated the expression of ET-1 and its receptors in the experimental autoimmune encephalomyelitis (EAE) model, a well-established murine model of CNS inflammation that recapitulates key aspects of multiple sclerosis (Carver and Didonna, 2023). This *in vivo* validation aims to determine whether the ET-1/ETRB axis is upregulated under neuroinflammatory conditions. These findings offer mechanistic insight into ET-1–mediated microglial activation and its potential relevance to neuroinflammatory processes.

## Materials and methods

### Cell culture

The human immortalize microglial cell line HMC3 (ATCC® CRL-3304™) was cultured in Eagle’s Minimum Essential Medium (EMEM; Gibco) supplemented with 10% fetal bovine serum and 1% penicillin–streptomycin at 37°C in a humidified atmosphere with 5% CO₂. For all stimulation experiments, cells were seeded in multi-well plates and treated with human endothelin-1 (ET-1, 100 nM) with or without the selective ETRB antagonist BQ788 (1 μM) or ETRA antagonist BQ123 (1 μM) for 24 h unless otherwise specified. Concentrations were selected based on prior studies of ET-1 signaling in glial cells (Koyama et al., 2013).

### RNA extraction and quantitative real-time PCR

Total RNA was prepared with 1 mL of TRIzol reagent (Invitrogen) according to the manufacturer’s instructions. The High-Capacity cDNA Reverse Transcription Kit (Applied Biosystems) was used to make 20 μL of cDNA from 2 μg of RNA. Gene expressions were analyzed using real-time PCR using TaqMan gene expression assay for EDN, EDNRA, EDNRB, NOS2, and GAPDH (Applied Biosystems) in a StepOne Plus from ABI. The ΔΔ cycle threshold method was used to determine mRNA levels. Gene expression was normalized to GAPDH levels.

### TNF-*α* and IL-6 ELISA

To determine the concentration of TNF-α and IL-6 in cell culture media of control or treated HMC3 cells, ELISA kits from R&D systems were used following the manufacturer’s instructions.

### Determination of ROS generation

ROS generation of HMC3 cells was measured by the Muse Oxidative stress kit using the Muse cell analyzer (Millipore, Billerica, MA, USA) fluorescent-based analysis. The manufacturer specific protocol was followed for the assay. In brief, HMC3 cells were treated with 100 nM ET-1 with or without BQ788 treatment and incubated for 24 h. 1 × 10^7^ cells/mL samples were prepared in 1X assay buffer and treated with Oxidative stress reagent, based on dihydroethidium (DHE) used to detect ROS that is oxidized with superoxide anion to procedure the DNA-binding fluorophore ethidium bromide which intercalates with DNA resulting in red fluorescence.

### Measurement of nitric oxide levels

Nitric oxide levels in the culture supernatant were evaluated by measuring NO_2_, a major stable product of nitric oxide. Briefly, 50 μL of each supernatant was mixed with an equal amount of the Griess solution (0.1% N-(1-naphthyl) ethyl-enediamine and 1% p-aminobenzenesulfonamide in 5% ortho-phosphoric acid) at room temperature for 30 min. Absorbance was read at 540 nm using a microplate spectrophotometer. Sodium nitrite was used for the standard curve.

### Detection of STAT-1 activation

The signal transducer and activator of transcription 1 (STAT-1) activation in HMC3 cells were measured by the Muse® STAT1 Activation Dual Detection Kit using the Muse cell analyzer (Millipore, Billerica, MA, USA). The manufacturer specific protocol was followed for the assay. In brief, HMC3 cells were treated with 100 nM ET-1 with or without BQ788 treatment and incubated for 24 h. Cells were fixed for 5 min and then permeabilized for 5 min. The antibody cocktail was added to the samples and incubated at room temperature and dark for 30 min. Samples were analyzed using the Muse cell analyzer.

### Mice EAE induction

Brain tissue samples from C57BL/6 mice with experimental autoimmune encephalomyelitis (EAE) (*n* = 6) and age-matched controls (*n* = 6) were obtained from a previously completed and IACUC-approved study from Hooke Laboratories (MA, USA). Total RNA was extracted from whole-brain homogenates, and gene expression levels of Edn1, Ednra, and Ednrb were assessed via RT-qPCR as described above. GAPDH was used as the housekeeping gene.

The sample size (*n* = 6 per group) was determined based on prior published studies using similar RT-qPCR analysis in EAE models to detect gene expression changes with sufficient power (Al-Ani et al., 2021). While formal power calculations were not performed, this sample size is commonly accepted for detecting statistically meaningful transcriptional differences in preclinical neuroinflammation research.

### ET-1 ELISA

Tissue samples were homogenized in the cold using a Bullet Blender Storm 24 (Next Advance) in RIPA containing protease inhibitors (Roche) at a 10:1 ratio of buffer volume to tissue wet weight. The homogenate was centrifuged at 17,000 g for 20 min at 4°C, and the supernatant was removed and saved. To determine the concentration of ET-1 in mice tissue, an ELISA kit from Invitrogen was used following the manufacturer’s instructions.

### Statistical analysis

Data are presented as mean ± SD from at least three independent experiments. Statistical comparisons were performed using one-way ANOVA followed by Tukey’s *post hoc* test or Student’s t-test where appropriate. A *p*-value of <0.05 was considered statistically significant. Graphs were generated using GraphPad Prism 10.

All experiments were performed using *in vitro* cell culture assays. Blinding was not applied during sample treatment or data collection. However, quantitative analyses were performed using standardized software settings to reduce investigator bias.

## Results

### ET-1 induces oxidative responses in human microglia cells

**Figure 1:**
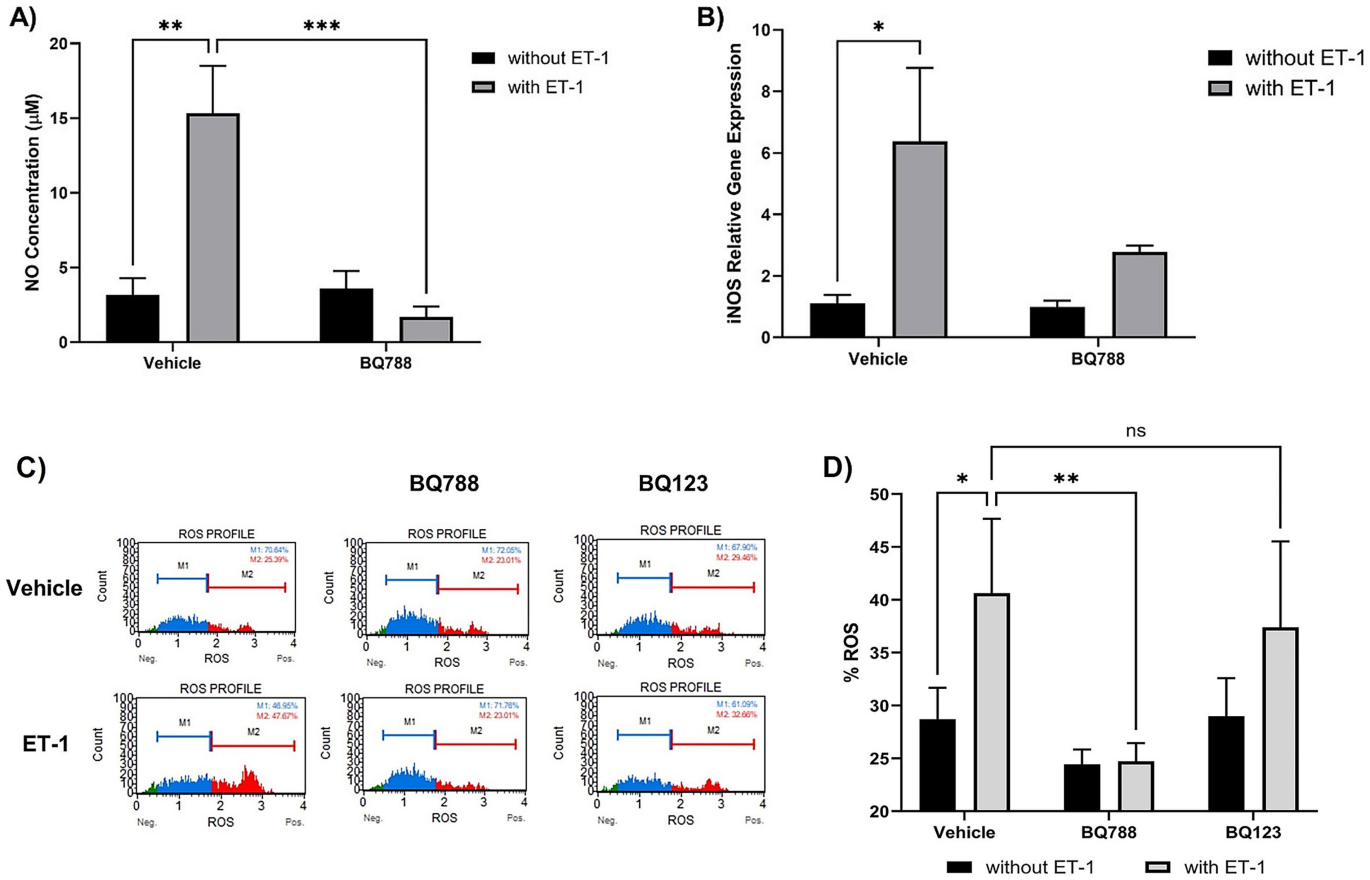
ET-1 induces oxidative responses by HMC3 cells. Microglia cells were treated with 100 nM ET-1 in the absence and presence of BQ788 (1 μM) for 24 h. **(A)** NO levels were measured using Griess Reagent. ET-1 treatment increases levels of NO compared to vehicle (*p* < 0.01, *n* = 4). Co-treatment with ET-1 and BQ788 results in significantly lower NO production compared to ET-1 alone (*p* < 0.001, *n* = 4). **(B)** RNA was extracted from microglia cells treated with ET-1 in the absence and presence of BQ788. Gene expression was quantified by qRT-PCR using a Taqman iNOS probe. **(C,D)** HMC3 cells were treated with ET-1 (100 nM) in the presence or absence of BQ788 (1 μM) or BQ123 (1 μM). Only BQ788 significantly attenuated ROS levels, while BQ123 did not reduce ET-1–induced oxidative responses. Data represent mean ± SD (*n* = 4). *p < 0.05; **p < 0.01; ***p < 0.001; ****p < 0.0001; ns, not significant.

To investigate the role of ET-1 in modulating oxidative stress in microglia, we stimulated HMC3 human microglial cells with ET-1 (100 nM) for 24 h. ET-1 treatment significantly increased nitric oxide (NO) production compared to untreated controls (*p* < 0.01), as measured by the Griess assay. Co-treatment with the selective ETRB antagonist BQ788 (1 μM) markedly reduced NO levels, indicating that ET-1–induced NO production is mediated through ETRB activation (Figure 1A). ET-1 also upregulated inducible nitric oxide synthase (iNOS) expression (Figure 1B), an effect reversed by BQ788 co-treatment, returning iNOS to basal levels.

Similarly, intracellular reactive oxygen species (ROS) were elevated following ET-1 exposure, as measured by DHE fluorescence (Figure 1C). ROS levels were significantly attenuated in cells co-treated with BQ788, supporting the involvement of ETRB in ET-1–induced oxidative responses (Figure 1D). In contrast, co-treatment with BQ123, a selective ETRA antagonist, did not significantly alter ROS levels in ET-1–treated cells (Figure 1D), suggesting a limited role for ETRA in these oxidative responses.

### ET-1 increases the secretion of Proinflammatory cytokines by human microglia cells

**Figure 2:**
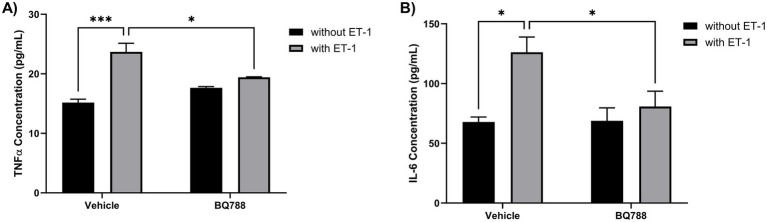
ET-1 increases the secretion of proinflammatory cytokines by HMC3 cells. HMC3 cells were incubated for 24 h in the absence or presence of 100 nM ET-1 with or without BQ788. Supernatants were collected to determine secretion levels of TNFα **(A)** and IL-6 **(B)** and measured by ELISA assay. Results represent the mean ± SD of *n* = 4. *p < 0.05; **p < 0.01; ***p < 0.001.

ET-1 stimulation also enhanced the proinflammatory profile of HMC3 cells. Human microglia cells treated with ET-1 showed an increase in TNFα concentrations when compared to controls (Figure 2A). Also, human microglia cells treated with ET-1 showed an increase in IL-6 concentrations when compared to controls (Figure 2B). These cytokine responses were blunted in the presence of BQ788, further confirming that ETRB signaling mediates ET-1–induced proinflammatory cytokine production. These results suggest that ET-1 promotes a classic inflammatory phenotype in human microglia.

### ET-1 activates STAT-1 pathway via ETRB

**Figure 3:**
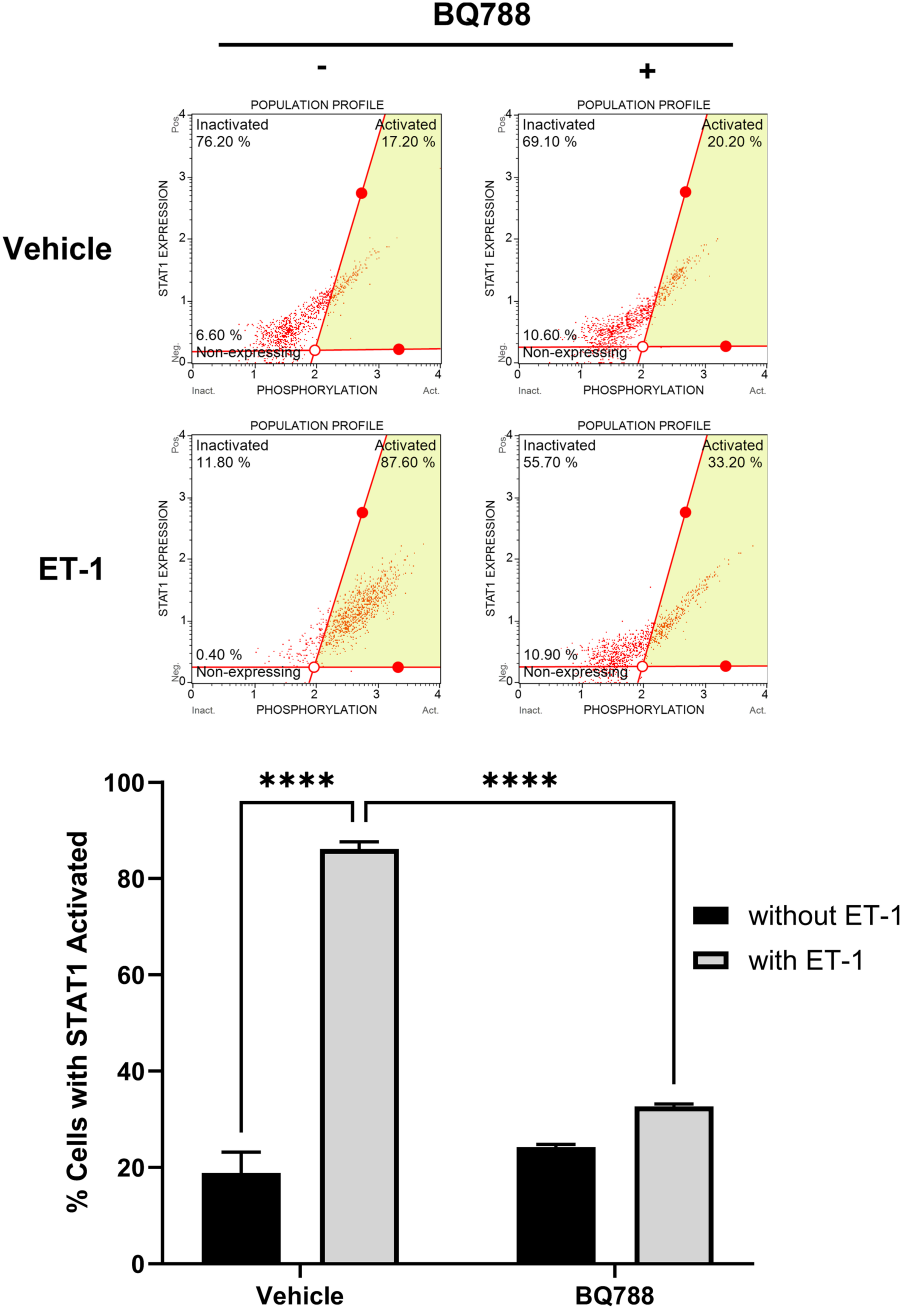
ET-1 activates the STAT-1 pathway. HMC3 cells were treated with 100 nM ET-1 with or without BQ788 treatment and incubated for 24 h. Samples were analyzed using a flow cytometry assay for p-STAT1. Results represent the mean ± SD of *n* = 4. *p < 0.05; **p < 0.01, ***p < 0.001; ****p < 0.0001.

To evaluate intracellular signaling events triggered by ET-1, we assessed STAT1 activation by flow cytometry. Phosphorylated STAT1 (Tyr701) levels were significantly increased in HMC3 cells treated with ET-1 compared to control (*p* < 0.01), while total STAT1 protein levels remained unchanged (Figure 3). Importantly, BQ788 treatment partially inhibited STAT1 phosphorylation, suggesting that ETRB is the receptor responsible for ET-1–induced STAT1 activation. This finding identifies the STAT1 pathway as a downstream effector of ET-1/ETRB signaling in microglia.

### ET-1 is overexpressed in EAE mice

**Figure 4:**
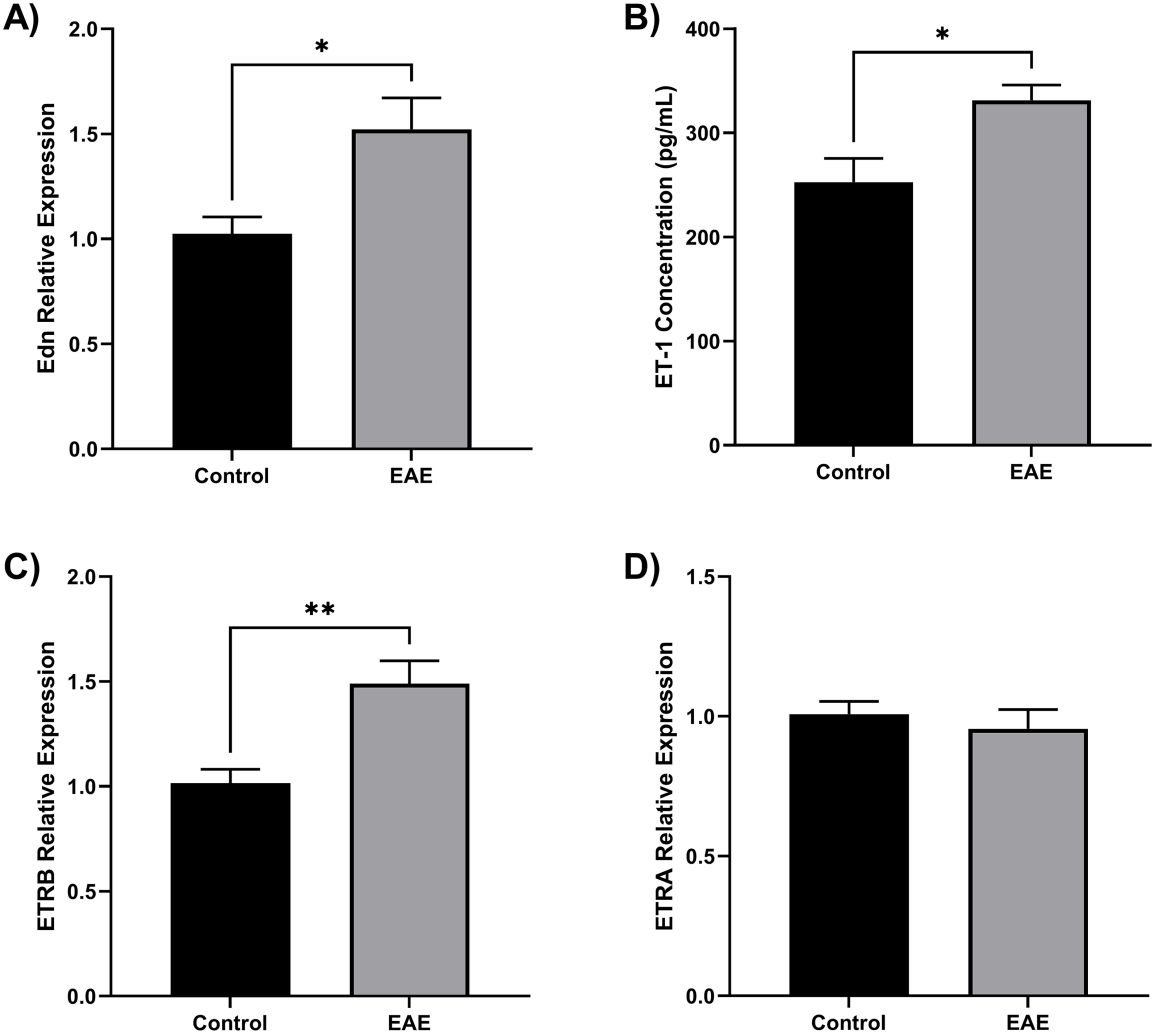
ET-1 is overproduced in EAE mice. Twelve female C57BL/6 mice were divided into EAE (*n* = 6) and control (*n* = 6). Brain tissue samples were homogenates for RNA and protein extraction. **(A)** Endothelin 1 gene (Edn) was overexpressed in the EAE brain compared to control (*p < 0.05). **(B)** Using ELISA, it was found that EAE brains have an elevated ET-1 concentration (*p < 0.05). **(C)** The ETRB gene was overexpressed in the EAE brain compared to the control (**p < 0.01). **(D)** The ETRA gene has no change in the EAE brain. Gene expression was quantified by qRT-PCR using Taqman Edn, Ednra, and Ednrb probes.

To determine whether ET-1 signaling components are upregulated in a model of CNS inflammation, we analyzed the expression of Edn1, Ednra, and Ednrb in brain tissue from mice with experimental autoimmune encephalomyelitis (EAE), a widely used model of neuroinflammation. RT-qPCR analysis of whole-brain homogenates revealed that Edn1 and Ednrb transcripts were significantly increased at the peak of disease compared to non-immunized controls (*p* < 0.05), while Ednra expression remained unchanged (Figure 4). ET-1 protein concentration was increased in EAE mice (Figure 4B). These data suggest that the ET-1/ETRB signaling axis is upregulated during neuroinflammation *in vivo*, supporting its potential role in mediating microglial activation in pathological states.

## Discussion

Microglia are central mediators of immune surveillance in the CNS, responding to injury and inflammation by producing a range of proinflammatory mediators and reactive species (Colonna and Butovsky, 2017; Kempuraj et al., 2016; Brown and Vilalta, 2015). In this study, we demonstrate that ET-1, a potent vasoactive peptide, also functions as a direct activator of human microglia. Specifically, we found that ET-1 stimulation increases NO and ROS production, upregulates proinflammatory cytokines TNF-*α* and IL-6, and activates the STAT1 signaling pathway. These effects were significantly attenuated by the selective ETRB antagonist BQ788, suggesting a receptor-specific mechanism. In contrast, blockade of ETRA with BQ123 had no significant effect on ET-1–induced oxidative responses, suggesting a limited role for ETRA in mediating microglial activation under these conditions.

Although ET-1 is well known for its vasoconstrictive and mitogenic properties, particularly through the ETRA (StatPearls, 2023), its engagement of the ETRB has been linked to glial reactivity and neuroinflammatory processes. The ETRB is highly expressed in astrocytes and endothelial cells (Koyama, 2021; Nie and Olsson, 1996), and its activation has been shown to modulate cytokine release, oxidative stress, and barrier integrity in the CNS (Koyama, 2021; Nie and Olsson, 1996; Faraco et al., 2013). However, its role in microglial biology has remained unexplored. Our findings fill this gap by identifying a functional link between ET-1/ETRB signaling and microglial activation.

The observed increase in NO and ROS following ET-1 stimulation is consistent with earlier findings in other CNS cell types (D’Antoni et al., 2017). ET-1 has been shown to enhance ROS generation in astrocytes, endothelial cells, and renal tissues, largely via NADPH oxidase activation (Schinelli, 2002; Faraco et al., 2013; Banecki and Dora, 2023). Previously, it was demonstrated that circulating ET-1 can impair cerebral microcirculation by increasing ROS levels and compromising endothelial function (Faraco et al., 2013). Our results extend these observations to microglia, suggesting that ET-1 may contribute to oxidative neuroinflammation through direct microglial engagement.

In parallel, we observed an increase in TNF-*α* and IL-6 production levels, two key cytokines implicated in neurodegeneration and neuroinflammatory priming (Ortí-Casañ et al., 2023; Jin et al., 2024; Pons-Espinal et al., 2024). This result supports previous studies showing that ET-1 can upregulate inflammatory gene expression through ETRB in astrocytes and peripheral immune cells (Koyama, 2021; Nie and Olsson, 1996; Kawanokuchi et al., 2006). The addition of STAT1 phosphorylation in our dataset further strengthens the case for ET-1 as a key signaling molecule in glial activation. STAT1 is a transcription factor classically involved in interferon signaling and is activated during M1-like proinflammatory polarization of microglia (StatPearls, 2023; Qin et al., 2012; Imitola et al., 2023). Its activation by ET-1 has been reported in renal epithelial and vascular cells (Schinelli, 2002; Stow et al., 2011), but this is the first study to demonstrate STAT1 engagement in human microglia following ET-1 exposure.

Furthermore, our analysis of whole-brain tissue from EAE mice revealed increased expression of Edn1 and Ednrb at the peak of disease, suggesting that the ET-1/ETRB axis is upregulated during CNS inflammation. These findings align with previous reports showing that ET-1 levels are elevated in inflammatory and demyelinating CNS conditions, including EAE and multiple sclerosis (Guo et al., 2014; Shin et al., 2001; Hostenbach et al., 2020). ETRB activation has been linked to proinflammatory cytokine release, astrocyte reactivity, and disruption of blood**–**brain barrier integrity (Koyama, 2021). While the use of bulk brain tissue limits precise cell-type attribution, the concurrent upregulation of ET-1 and ETRB supports the hypothesis that this pathway contributes to CNS inflammation. Our *in vivo* data thus complement the *in vitro* findings by establishing that microglia are likely to encounter elevated ET-1 levels in inflamed brain environments, further implicating the ET-1/ETRB axis as a modulator of neuroinflammation.

This study has several limitations. First, we relied on the use of HMC3 immortalized human microglial cell line, which may not fully replicate the transcriptional and functional diversity of primary human microglia (Colonna and Butovsky, 2017; Janabi et al., 1995). Although HMC3 cells provide a useful model for mechanistic assays, future validation using primary human microglia or *in vivo* models is necessary. Second, our analysis was restricted to acute time points following ET-1 stimulation. Longer exposures may reveal additional downstream effects such as sustained cytokine production, morphological changes, or neurotoxic behaviors. Third, we did not assess microglial polarization markers (e.g., CD86, CD206) or phagocytic function, which would further characterize the phenotype induced by ET-1. Fourth, although we included a single-dose BQ123 control to assess the role of ETRA, we did not perform a full dose–response analysis to generate an IC50 curve. While prior studies support the effectiveness of the 1 μM dose in vitro, we acknowledge that further dose-dependent evaluations could strengthen conclusions regarding ETRA’s limited role. Fifth, though our findings demonstrate increased STAT1 phosphorylation following ET-1 exposure, we did not perform loss-of-function or inhibition experiments to functionally validate STAT1’s role in mediating downstream effects. Future studies are warranted to dissect the mechanistic contribution of STAT1 signaling to ET-1–induced proinflammatory responses in microglia. Additionally, while the EAE model provides translational context, further studies using cell-type-specific isolation or immunohistochemistry are needed to confirm microglial involvement *in vivo*.

Nonetheless, this study makes a significant contribution by establishing ET-1 as a direct activator of microglial oxidative and inflammatory responses through ETRB and STAT1 signaling. It expands the traditional view of ET-1 as a vascular modulator, suggesting a more complex role in CNS immune regulation. These findings are particularly relevant given the increasing recognition that neuroinflammation contributes to a wide range of neurological disorders, from neurodegeneration to cerebrovascular disease (Kempuraj et al., 2016; Brown and Vilalta, 2015; Carver and Didonna, 2023; Zhao et al., 2022).

In conclusion, our results demonstrate that ET-1 induces oxidative and inflammatory activation of human microglia via the ETRB and STAT1 signaling. These responses may contribute to broader neuroinflammatory cascades in the CNS. The upregulation of Edn1 and Ednrb in EAE brain tissue suggests that this pathway is active *in vivo* during neuroinflammatory episodes. Targeting the ET-1/ETRB axis may therefore represent a promising strategy for modulating microglia-driven inflammation in CNS disorders. Future studies should explore this mechanism in greater detail using primary microglial models and assess its relevance across different neurological disease contexts.

## Data Availability

The datasets presented in this study can be found in online repositories. The names of the repository/repositories and accession number(s) can be found below: Mendeley Data, http://dx.doi.org/10.17632/2gtcvmf4t2.1.
